# Divergent dysregulation of gene expression in murine models of fragile X syndrome and tuberous sclerosis

**DOI:** 10.1186/2040-2392-5-16

**Published:** 2014-02-24

**Authors:** Sek Won Kong, Mustafa Sahin, Christin D Collins, Mary H Wertz, Malcolm G Campbell, Jarrett D Leech, Dilja Krueger, Mark F Bear, Louis M Kunkel, Isaac S Kohane

**Affiliations:** 1 Informatics Program, Boston Children’s Hospital, Harvard Medical School, Boston, MA, USA; 2 The F.M. Kirby Neurobiology Center, Department of Neurology, Boston Children’s Hospital, Harvard Medical School, Boston, MA, USA; 3 Howard Hughes Medical Institute, Department of Genetics, Boston Children’s Hospital, Harvard Medical School, Boston, MA, USA; 4 Howard Hughes Medical Institute, The Picower Institute for Learning and Memory, Department of Brain and Cognitive Sciences, Massachusetts Institute of Technology, Cambridge, MA, USA; 5 Center for Biomedical Informatics, Harvard Medical School, Boston, MA, USA

**Keywords:** Fragile X syndrome, Tuberous sclerosis, Autism, Cerebellum, Blood, Gene expression, Murine model

## Abstract

**Background:**

Fragile X syndrome and tuberous sclerosis are genetic syndromes that both have a high rate of comorbidity with autism spectrum disorder (ASD). Several lines of evidence suggest that these two monogenic disorders may converge at a molecular level through the dysfunction of activity-dependent synaptic plasticity.

**Methods:**

To explore the characteristics of transcriptomic changes in these monogenic disorders, we profiled genome-wide gene expression levels in cerebellum and blood from murine models of fragile X syndrome and tuberous sclerosis.

**Results:**

Differentially expressed genes and enriched pathways were distinct for the two murine models examined, with the exception of immune response-related pathways. In the cerebellum of the *Fmr1* knockout (*Fmr1-*KO) model, the neuroactive ligand receptor interaction pathway and gene sets associated with synaptic plasticity such as long-term potentiation, gap junction, and axon guidance were the most significantly perturbed pathways. The phosphatidylinositol signaling pathway was significantly dysregulated in both cerebellum and blood of *Fmr1*-KO mice. In *Tsc2* heterozygous (+/−) mice, immune system-related pathways, genes encoding ribosomal proteins, and glycolipid metabolism pathways were significantly changed in both tissues.

**Conclusions:**

Our data suggest that distinct molecular pathways may be involved in ASD with known but different genetic causes and that blood gene expression profiles of *Fmr1-*KO and *Tsc2+/−* mice mirror some, but not all, of the perturbed molecular pathways in the brain.

## Background

Autism spectrum disorder (ASD) manifests significant heterogeneity in part because of the interaction of underlying genetic [1-3], neurobiological, and environmental factors [4,5] during early brain development. This heterogeneity presents one of the main obstacles to the development of effective treatments for ASD. The complex genetics of ASD suggest that it is a large set of related disorders with diverse mechanisms; however, many of the etiologies implicated in ASD may converge on a few common pathways. Further research on single gene disorders associated with ASD such as tuberous sclerosis complex (TSC) and fragile X syndrome (FXS) may lead to an understanding of common dysfunction at the cellular or circuit level for a majority of ASD. In a recent survey of over 14,000 individuals under age 35 with ASD in a Boston area hospital, Kohane and colleagues reported that the prevalence of genetic disorders of FXS and TSC in individuals with ASD were 0.5% and 0.8% [6]. Conversely, 30% and 50-61% of patients with FXS and TSC present ASD core symptoms, respectively [7,8]. If such shared pathophysiology exists, then treatments developed for a target in one disorder might be applicable to others. Mouse models for ASD serve an increasingly important role in providing a pre-clinical test of promising pharmacological therapeutics [9,10]. Inactivating mutation in *Tsc2* (*Tsc2*+/− mice) showed defects in axon guidance [11] and cognitive deficits such as impaired water maze performance [12], and mice with *Fmr1*-knockout (KO) presented impairments in long-term depression, hyperactivity, anxiety-like, and unusual social behaviors [13]. Therefore, determining the degree to which there are shared molecular mechanisms in these models will inform clinical trials, particularly those that address populations with genetically heterogeneous causes of ASD.

Although several cellular mechanisms may be implicated (reviewed in Fatemi et al. [14]), accumulating data support a role for the PI3K-mTOR signaling cascade in several genetic causes of ASD. Evidence for the PI3K-mTOR pathway first emerged from TSC [15,16] and mutations in the *PTEN* gene associated with ASD and macrocephaly [17-19]. Later, investigation of copy number variants (CNV) in autistic individuals identified that PI3K-mTOR pathway-related genes were located in CNV hotspots [20]. These findings have led to the hypothesis that overactivation of the mTOR pathway could lead to abnormal synaptic function owing to an excess of protein synthesis at the synapse [21]. Genetic evidence that directly implicates a translation initiating factor, EIF4E, which is a downstream target of mTOR, in ASD has provided further support for this hypothesis [22]. Interestingly, exposure to teratogens such as valproate *in utero* can lead to ASD in children [23], and valproate can also modulate this signaling pathway [24], suggesting that environmental factors associated with ASD can also play a role in PI3K-mTOR pathway regulation [25]. More recently, studies have found that PI3K-mTOR signaling is upregulated in mouse models of FXS, one of the most common genetic causes of ASD [26-28].

Together, the aforementioned findings suggest that an upregulated PI3K-mTOR signaling cascade might be a common mechanism in ASD and therefore would potentially be a promising drug target. Indeed, clinical trials using inhibitors of mTOR are already in progress in patients with TSC. We hypothesized that if the PI3K-mTOR signaling pathway is dysregulated in various causes of ASD, then these disorders should present with a similar gene expression profile signature. We chose to analyze TSC and FXS, two Mendelian disorders highly associated with ASD. Better understanding of similarities and differences of the cellular and molecular defects leading to abnormal neurological function in these two disorders is essential to the development of new therapies for ASD. Here, we used mouse models available for both genetic disorders to investigate the similarities and differences between gene expression profiles in the brain and blood cells.

## Methods

### Murine models of fragile X syndrome and tuberous sclerosis

To identify molecular signatures of each mouse model of ASD, we performed gene expression profiling on cerebellum and peripheral blood collected from two mouse models and compared to wild-type (WT) controls. We used cerebellum where the most consistent abnormalities were reported in the patients with ASD [14]. Post-mortem studies have shown a reduced number of Purkinje cells (PC), and several neuroimaging studies reported enlarged cerebella in ASD [29,30]. The cerebellum is also implicated in social interaction [31], and the loss of *Tsc1* from cerebellar PC was associated with autistic-like behaviors [32]. Additionally, we profiled whole blood from the same individual mouse to compare with the gene expression changes in cerebellum.

All male C57BL/6 congenic *Fmr1*-KO mice and *Tsc2+/−* mice with mixed 129/SvJae-C57BL/6 J background have been previously described [33,34]. We profiled *Tsc2+/−* mice since homozygous *Tsc2* KO was embryonic lethal. The mice were killed at 8–10 weeks of age following the institutional animal care and use committee (IACUC) euthanasia criteria (the Boston Children’s Hospital IACUC animal protocol no. 12-07-2227R). For the *Fmr1*-KO model, 5 KO and 5 WT mice were profiled, and for the *Tsc2+/−* model 3 transgenic and 3 WT mice were profiled. Paired blood and cerebellum samples were prepared for gene expression profiling.

### Genome-wide gene expression profiling using microarrays

A total of 250 ng RNA was processed using established Affymetrix protocols for the generation of biotin-labeled cRNA, and the hybridization, staining, and scanning of arrays were performed. Briefly, total RNA was converted to double-stranded cDNA using a T7 primer and biotin-labeled cRNA was then generated from the cDNA by in vitro transcription. The cRNA was quantified (using A260) and fragmented. Fragmented cRNA was hybridized to the Affymetrix Mouse Gene ST 1.0 array and scanned on an Affymetrix GeneChip scanner 3000 at 2.5 μm resolution [35]. Microarray data are available at the Gene Expression Omnibus database (GSE40630).

### Validation of gene expression changes using quantitative RT-PCR

Total RNA was extracted using TRIzol according to the manufacturer’s instruction. The RNA amount was measured using the Nanodrop (Thermo Scientific); 100 ng of total RNA was reversed transcribed using a cDNA reverse transcription kit with random primers (Applied Biosystems). SyBr Green PCR Master Mix (Applied Biosystems) was used to amplify and detect signals from cDNA with 1 mM gene-specific primers. Amplified signals were collected by the 7300HT Fast Real-Time System (Applied Biosystems) and normalized to *Gapdh*. Primer sequences used for this study were *Gapdh* forward 5′-tgtgtccgtcgtggatctga-3′ reverse 5′-cctgcttcaccaccttcttga-3′, *Fmr1* forward 5′-ggtcaaggaatgggtcgagg-3′, reverse 5′-agtcgtctctgtggtcagat-3′, *Tsc2* forward 5′-cagtgtcgaccagctgtctt-3′, reverse 5′-tcacgctgtctggtcttgtc-3′, *Eps811* forward 5′-cagctacaacacgagaagcg-3′, reverse 5′-ccgaaccttccaccatttgc-3′ and *Grin3a* forward 5′-ctgaaacctgggtgtgaggt-3′, and reverse 5′-aatgctgttcccacacaaca-3′.

### Microarray analysis

All microarrays were normalized together at the probe-level using a quantile method, and the Affymetrix Probe Logarithmic Intensity ERror (PLIER) model was used to calculate the absolute gene expression levels as previously described [35]. We fitted a linear model of the tissue (i.e., blood vs. cerebellum) and treatment (i.e., transgenic vs. WT) as predicting variables to each probe set. Two murine models were analyzed separately as different background strains were used. Differentially expressed genes in each murine model were compared to the differentially expressed genes between the wild types of two models. The false discovery rate (FDR) was calculated using Storey and Tibshirani’s method [36]. We did not use non-parametric tests such as the Wilcoxon rank sum test because of the granularity of the test statistics with a small number of samples per group.

We identified enriched pathways using the Gene Set Enrichment Analysis (GSEA) [37]. The Kyoto Encyclopedia of Genes and Genomes (KEGG) pathways of size 15–500 were used for pathway analysis. Due to the relatively small number of samples in each group, we randomly sampled gene sets of equal size for each KEGG pathway to calculate the background distribution of enrichment scores. This procedure was done with 2,000 random drawings, thus the minimum permutation p-value was 0.0005. We used permutation *p*-value 0.05 as the significance threshold for GSEA, and corresponding FDRs were described. To identify the core set of genes that accounts for the gene set’s enrichment signal, we used leading edge analysis as described in Subramanian et al. [37] where a leading edge subset was defined as the subset of genes that gave the maximum enrichment score. These genes were the topmost correlated genes with phenotype in a gene set.

To compare differentially expressed genes with the list of known ASD candidate genes as curated in the SFARIgenes 2.0 database (http://gene.sfari.org/) [38], we mapped mouse genes to human homologs using the Mouse Genome Informatics (MGI) Web database (http://www.informatics.jax.org) and performed hypergeometric tests to check the significance of overlap.

## Results

### Distinct gene expression changes define Fmr1 and Tsc2 transgenic models

**Figure 1:**
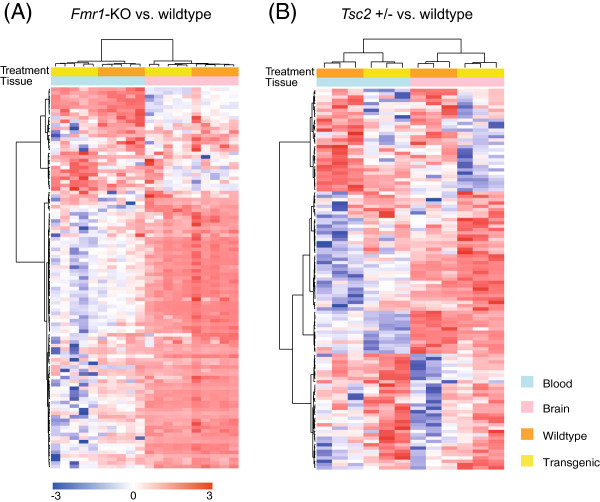
**Agglomerative hierarchical clustering of blood and brain samples. (A)** Hierarchical clustering of *Fmr1*-KO and wild-type samples. One hundred seven differentially expressed transcripts (nominal *p*-value < 0.01) are used for hierarchical clustering of transcripts (*rows* in the heatmap) and samples (*columns* in the heatmap). The gene expression levels are normalized across samples, and *red* (upregulated) and *blue* (downregulated) are color coded according to the *bottom color bar. Color keys* on the top of the heatmap denote tissue type and transgenic model. By far the most pronounced clustering is by tissue type. Within the blood samples, the two mice strains are clearly separated. **(B)** Hierarchical clustering of *Tsc2* +/− and wild-type samples. One hundred fifteen significant transcripts are used for cluster analysis. Two mice strains formed separate clusters in each tissue type. The tissue specificity is not significant for the differentially expressed transcripts in *Tsc2* +/− mice, whereas a majority of differentially expressed genes are highly expressed in cerebellum of *Fmr1*-KO mice (*lower right cluster* in the heatmap of Figure 1A).

A total of 107 and 115 probe sets were significantly changed in *Fmr1*-KO and *Tsc2+/−* mice compared to corresponding WT littermates, respectively (uncorrected *p*-value < 0.01). Not surprisingly, *Fmr1* was the most significantly downregulated gene in *Fmr1*-KO (*p*-value 8.85 × 10^-6^, corresponding FDR 0.29). We used nominal *p*-values estimated from a linear model less than 0.01 to rank significant probe sets because multiple testing correction procedures did not make any gene significant. The expression levels of 16 out of 107 significant probe sets (15.0%) in *Fmr1*-KO mice and 58 out of 115 significant probe sets (50.4%) in *Tsc2+/−* mice did not show a significant difference between blood and brain. We used an agglomerative hierarchical clustering with the significant probe sets for each model to explore the similarity of gene expression profiles in two genotypes and across tissue types. Samples were clearly separated by tissue type and then by genotype (Figure 1A and 1B). Interestingly, 71% of significant probe sets in *Fmr1*-KO mice were highly expressed in cerebellum compared to blood (Figure 1A). For the *Tsc2+/−* model, the average expression levels of 63 out of 115 probe sets (57.5%) were higher in cerebellum (Figure 1B). Differentially expressed genes with statistical scores are listed in Additional file 1: Table S1 (*Fmr1-*KO vs. WT) and Additional file 1: Table S2 (*Tsc2+/−* vs. WT).

We compared the gene expression profiles of WT mice since *Tsc2+/−* mice had 129/SvJae-C57BL/6 J backgrounds compared to C57BL/6 congenic backgrounds of *Fmr1*-KO mice. A total of 1,486 probes sets were differentially expressed between two WT strains. Seven probe sets, representing five genes, *Cog7, Cc2d1a, Smurf1, Sec31a*, and *AU040320*, overlapped with the differentially expressed genes in *Fmr1*-KO vs. WT comparison. For the differentially expressed genes in *Tsc2+/−* vs. WT comparison, 13 probe sets (representing 9 genes: *Fhad1, Pgam5, Cts8, Piwil4, Tmem101, Heatr7a, Mucl1, 2210404J11Rik*, and *Fam181b*) were significantly different between two WT mice strains.

Epidermal growth factor (EGF) receptor pathway substrate 8-like 1 *(Eps811)* (Affymetrix probe set ID: 10549655) was the only gene that was significant in both murine models. *Eps811* was upregulated in Tsc2+/− mice, but downregulated in *Fmr1-*KO mice. Post hoc two-group comparison of the transgenic model to WT for each tissue showed that *Eps811* was upregulated in blood (Welch’s *t*-test *p*-value 0.07) and brain (Welch’s *t*-test *p*-value 0.06) of Tsc2+/− mice, while it was downregulated in blood (Welch’s *t*-test *p*-value 0.015) of *Fmr1-*KO mice. Downregulation of *Eps811* in brain of *Fmr1-*KO was not significant (Welch’s *t*-test *p*-value 0.38). The homolog of *Eps811* in human, *EPS8L1* is a member of EPS8-related proteins that play an important role in actin remodeling in response to EGF [39], and *EPS8* is one of the reported targets of FMRP [40]. Interestingly, Stamatakou and colleagues reported that Eps8 loss of function impaired the structural and functional plasticity of synapses induced by long-term potentiation in primary rat hippocampal neurons [41]. Phenotypically, *Eps8-*KO mice have impaired learning and memory, and excessive synaptic growth and abnormal spine morphology were observed in the hippocampus [42]. In human samples, the average expression of *EPS8* in fusiform gyri was significantly lower among the patients with ASD compared to controls [42]. Although *Eps8* itself was not significantly changed in our study, *EPS8* family genes are interesting candidates for further investigation. Thus, we performed qRT-PCR of *Eps8* in brain samples of two murine models.

Of the differentially expressed genes, several were also found in the expert curated database of autism candidate genes. Of these, *Chd7, Fmr1*, and *Tmlhe* were differentially expressed in *Fmr1*-KO mice, and *Oxtr* and *Taf1c* were significantly changed in *Tsc2+/−* mice. The differentially expressed genes were not significantly enriched for known ASD candidate genes in human (hypergeometric test *p*-value 0.48 for *Fmr1-*KO and 0.70 for *Tsc2+/−*).

### Validation of differentially expressed genes using quantitative RT-PCR

Quantitative RT-PCR for individual genes was used to further confirm the results of the expression profiling in the brain samples used for the initial analysis. As expected, *Fmr1* gene expression was significantly decreased in *Fmr1*-KO mice as compared to controls (average 5.24-fold downregulated, Welch’s *t*-test *p*-value 0.025), while *Fmr1* was unchanged in *Tsc2*+/− mice (Welch’s *t*-test *p*-value 0.70). *Tsc2* gene expression showed a trend of decreased expression in both *Tsc2*+/− and *Fmr1*-KO mice as compared to WT mice that did not reach significance (average fold change 1.12 and 1.31 downregulation, Welch’s *t*-test *p*-value 0.55 and 0.27, respectively). We previously found a significant reduction of Tsc2 protein in cortical neurons of *Tsc2+/−* mice [11]; however, *Tsc2* mRNA expression was not significantly downregulated. Further, our results confirm that *Eps8l1* exhibited a three-fold increase in *Tsc2*+/− animals as compared to WT mice (average 3.15-fold upregulation, Welch’s *t*-test *p*-value 0.06). However, *Eps8l1* expression shows a decreased trend—an average 1.26 fold downregulation—in *Fmr1*-KO compared to WT similar to that observed in the expression profiling but not significantly changed (Welch’s *t*-test *p*-value 0.74).

We observed significant downregulation of subtype 3a of the N-methyl-D-aspartate receptor gene (*Grin3a*) in the blood of *Fmr1*-KO mice (uncorrected *p*-value 0.0098). *Grin3a* was not significant in brain with microarray data; however, a quantitative RT-PCR analysis of the same samples showed a significant downregulation of this gene in *Fmr1*-KO brain (5.69-fold downregulated, Welch’s *t*-test *p*-value 0.046).

### Enriched biological pathways in blood and brain of the two mouse models

We explored whether similar sets of biological pathways were perturbed in both models using GSEA [37] as there was only one overlapping gene—*Eps8l1*—between the two lists of differentially expressed genes in *Fmr1*-KO and *Tsc2+/−* mice. The genes that contributed to making a pathway significant were identified using leading edge analysis. First, all genes were ranked by a per-gene signal-to-noise ratio that was defined as mean difference divided by the sum of standard deviation of each group. Then a running sum was calculated for each gene set. Beginning with the top-ranked gene, the running sum increased when a gene in a gene set was found and decreased otherwise. The enrichment score (ES) was defined to be the largest value of the running sum, and the genes that maximized ES were defined as the leading edge subset.

Two pathways related to cytokine and complement-mediated signaling, cytokine-cytokine receptor interaction (HSA04060, FDR < 0.0005) and complement and coagulation cascades (HSA04610, FDR < 0.0005), were the most significantly enriched pathways in *Fmr1*-KO brain (Table 1). Neuroactive ligand receptor interaction (HSA04080, FDR < 0.0005), long-term potentiation (HSA04720, FDR 0.0023), gap junction (HSA04540, FDR 0.0429), and axon guidance (HSA04360, FDR 0.048) were also enriched in *Fmr1*-KO brain. Among the signaling pathways, the PI3K signaling pathway (HSA04070) was changed in both brain (FDR 0.042) and blood (FDR 0.05) of *Fmr1*-KO mice. Eight genes were in the leading edges of the two tissues (see Methods). These were *Dgkb, Dgkg, Dgkh, Inpp4b, Inpp5a, Itpr3, Plcb4,* and *Prkca*. Glutamate metabolism (HSA00251) was also enriched in *Fmr1*-KO brain (FDR 0.043).

**Table 1:** **Enriched pathways in blood and brain of *Fmr1 *** **knockout mice**

**KEGG categories**	**Name**	**SIZE**	**NES**	**NOM p-val**	**FDR q-val**
***Brain***					
Nervous system	HSA04080 Neuroactive ligand receptor interaction	223	2.34	< 0.0005	0.0000
	HSA04720 Long-term potentiation	62	−2.15	< 0.0005	0.0023
	HSA04540 Gap junction	77	−1.75	< 0.0005	0.0429
	HSA04360 Axon guidance	125	−1.81	< 0.0005	0.0480
Immune system	HSA04060 Cytokine-cytokine receptor interaction	212	2.72	< 0.0005	< 0.0005
	HSA04610 Complement and coagulation cascades	47	2.32	< 0.0005	< 0.0005
	HSA04650 Natural killer cell mediated cytotoxicity	95	1.70	0.0015	0.0379
	HSA04612 Antigen processing and representation	42	1.67	0.0049	0.0397
	HSA04640 Hematopoietic cell lineage	66	2.09	< 0.0005	0.0004
Signaling pathways	HSA01430 Cell communication	118	2.14	< 0.0005	0.0004
	HSA04630 Jak-STAT signaling pathway	137	2.07	< 0.0005	0.0005
	HSA04070 Phosphatidylinositol signaling system	65	1.77	0.0029	0.0420
	HSA04910 Insulin signaling pathway	126	1.80	< 0.0005	0.0455
Metabolism	HSA00150 Androgen and estrogen metabolism	33	1.84	0.0043	0.0118
	HSA00590 Arachidonic acid metabolism	39	1.81	0.0017	0.0147
	HSA00361 γ-hexachlorocyclohexane degradation	17	1.69	0.0118	0.0370
	HSA00020 Citrate cycle	25	1.93	0.0023	0.0375
	HSA00592 α-linolenic acid metabolism	15	1.70	0.0127	0.0392
	HSA00251 Glutamate metabolism	30	1.87	0.0038	0.0430
Folding, sorting, and degradation	HSA04120 Ubiquitin mediated proteolysis	36	1.78	0.0039	0.0453
	HSA04130 Snare interactions in vesicular transport	29	1.84	< 0.0005	0.0450
***Blood***					
Signaling pathways	HSA04070 Phosphatidylinositol signaling system	65	1.78	< 0.0005	0.0500

Eight pathways were significantly enriched in *Tsc2+/−* brain, while 11 pathways were enriched in *Tsc2+/−* blood (Table 2). Pathways associated with the immune system were significantly enriched in both blood and brain. Ribosome (HSA03010, FDR < 0.0005), cytokine-cytokine receptor interaction (HSA04060, FDR < 0.0005), and oxidative phosphorylation (HSA00190, FDR < 0.0005) were the most significant pathways in the brain, and the ribosome pathway was also significantly deregulated in *Tsc2+/−* blood (FDR 0.0468). In the *Tsc2+/−* brain, cytokine-cytokine receptor interaction (FDR < 0.0005) and hematopoietic cell linage (HSA04640, FDR 0.0389) were significant, while the Toll-like receptor signaling pathway (HSA04620, FDR 0.0016) and B-cell receptor signaling pathway (HSA04662, FDR 0.0072) were significant in *Tsc2+/−* blood. None of the differentially expressed genes between two WT strains was a member of significant pathways that we identified above.

**Table 2:** **Enriched pathways in blood and brain of *Tsc2+/− *** **mice**

**KEGG categories**	**Name**	**SIZE**	**NES**	**NOM p-val**	**FDR q-val**
***Brain***					
Immune system	HSA04060 Cytokine-cytokine receptor interaction	212	2.37	< 0.0005	< 0.0005
	HSA04640 Hematopoietic cell lineage	66	1.75	< 0.0005	0.0389
Signaling pathways	HSA04010 MAPK signaling pathway	241	−1.92	< 0.0005	0.0431
	HSA01430 Cell communication	118	2.15	< 0.0005	0.0007
Translation	HSA03010 Ribosome	66	3.21	< 0.0005	< 0.0005
Metabolism	HSA00563 Glycosylphosphatidylinositol anchor biosynthesis	21	−1.85	0.0017	0.0444
	HSA00190 Oxidative phosphorylation	110	2.75	< 0.0005	< 0.0005
	HSA00980 Metabolism of xenobiotics by cytochrome P450	35	1.77	< 0.0005	0.0379
***Blood***					
Nervous system	HSA05010 Alzheimer’s disease	26	1.92	< 0.0005	0.0034
Immune system	HSA04620 Toll-like receptor signaling pathway	94	2.00	< 0.0005	0.0016
	HSA04662 B cell receptor signaling pathway	60	1.85	< 0.0005	0.0072
Cell growth and death	HSA04110 Cell cycle	107	−2.31	< 0.0005	0.0005
	HSA04210 Apoptosis	73	1.78	0.0006	0.0181
Translation	HSA03010 Ribosome	66	1.68	0.0026	0.0468
Metabolism	HSA01032 Glycan structures degradation	29	2.16	< 0.0005	0.0000
	HSA00530 Aminosugars metabolism	29	1.95	0.0007	0.0035
	HSA00531 Glycosaminoglycan degradation	17	1.92	< 0.0005	0.0043
	HSA00511 Other glycan degradation	15	1.88	0.0007	0.0047
	HSA00600 Sphingolipid metabolism	31	1.69	0.0064	0.0473

### Common signature of *Fmr1* and *Tsc2* transgenic models

**Figure 2:**
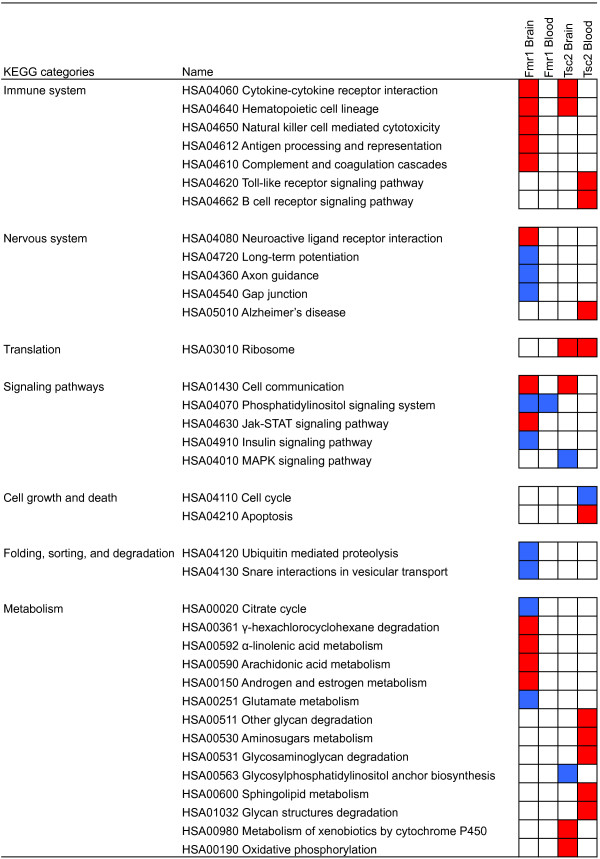
**Enriched pathways in *Fmr1*-KO and *Tsc2+/− *models of ASD, found using the Gene Set Enrichment Analysis (GSEA).***Red* (upregulated) and *blue* (downregulated) squares in the matrix represent enriched pathways for each data set (false discovery rate ≤ 0.05). Two immune system pathways (cytokine-cytokine receptor signaling pathway and hematopoietic cell lineage) and one signaling pathway (cell communication) were significant in the brain gene expression profiles of both mice models.

Cytokine-cytokine receptor interaction pathway, hematopoietic cell linage, and cell communication were enriched in brain gene expression profiles of both *Fmr1*-KO and *Tsc2+/−* mice (Figure 2). We performed leading edge analysis to find core genes that made a pathway significant, although each gene was not necessarily differentially expressed. For the cytokine-cytokine receptor pathway (N = 212), 105 and 106 genes were the core genes in *Tsc2+/−* and *Fmr1*-KO brain data sets. *Il7r* was the only gene that showed marginal significances in both data sets (post-hoc Welch’s *t*-test *p*-values 0.011 and 0.013 in *Tsc2+/−* and *Fmr1*-KO brain profiles, respectively). The same gene was also the only common significant gene for the hematopoietic cell lineage pathway. Thirty-two genes were in common between the leading edges of cell communication for *Fmr1*-KO and *Tsc2+/−* brain; however, no genes were significantly differentially expressed. Interestingly, *Tsc2* gene expression was downregulated in *Fmr1*-KO blood (post-hoc Welch’s *t*-test *p*-value 0.0045).

## Discussion

We hypothesized that *Fmr1*-KO and *Tsc2+/−* mice would have similar gene expression profiles. In contrast, our findings indicate that different gene expression signatures define these two monogenic mouse models of ASD. Global expression profiles of the two models examined were distinct, such that only one gene—*Eps8l1*—was in common. This is a particularly surprising result since translational dysregulation as well as aberrant synaptic protein synthesis associated with both disorders has been proposed as one possible pathway leading to autistic phenotypes, including cognitive impairment [21]. Our data indicate that FXS and TSC may have very distinct brain and blood cellular phenotypes despite the fact that both syndromes result in similar behavioral and cognitive symptoms.

Nonetheless, we did find that the cytokine and complement signaling pathways are differentially regulated in both mouse models although the specific genes affected within these pathways were different. The immune system has been implicated in ASD in multiple ways, but the exact mechanism of the interaction between the immune system and genetic disorders that result in an increased risk of autism has not been well studied. Modulation of the immune system may not be completely unexpected since the TSC-mTORC1 pathway regulates inflammatory responses after bacterial stimulation in monocytes, macrophages, and primary dendritic cells [43], and this pathway contributes to cytokine upregulation in response to endotoxins [44]. However, this is of particular interest in light of a recent study that showed that immune activation during gestation can markedly worsen the neurological phenotype of *Tsc2+/−* mouse pups [45]. Similarly, preliminary observations indicate that plasma protein levels of a number of cytokines differ between individuals with and without FXS. Furthermore, differences in cytokine and other immune-signaling genes were observed between the FXS group with autism and the FXS group without autism [46]. On the other hand, Yuskaitis and colleagues investigated the peripheral immune system of *Fmr1*-KO mice, but did not find any differences in either the T-cell population at basal and stimulated status or the proinflammatory cytokines TNFα and IFNγ at basal and stimulated status [47]. How the loss of FMRP leads to changes in the immune system, however, remains unclear.

Accumulating evidence over the last few years indicates that the TSC and FMRP pathways interact. However, precisely how these pathways interact remains an open question. On the one hand, FMRP can be phosphorylated by S6K, an enzyme downstream of TSC [48]. On the other hand, mTOR, the kinase inhibited by the TSC2 protein, has increased activity in *Fmr1*-KO neurons [26], and FMRP-deficient cells display increased activity of PI3K, an enzyme upstream of TSC proteins [27]. Recently Auerbach et al. reported that the synaptic dysfunction in the CA1 region of the hippocampus of *Tsc2+/−* mice was opposite to that of *Fmr1*-KO mice [49]. In fact, manipulating the mGluR receptors with positive allosteric modulators was sufficient to rescue this defect in *Tsc2+/−*, while inhibiting the mGluR receptors was necessary in the case of *Fmr1*-KO mice. Finally, a genetic cross of the *Tsc2+/−* and *Fmr1*-KO mice was similar to WT in CA1 synaptic physiology and contextual learning. These results argue that loss of Tsc2 and Fmr1 have some opposite cellular phenotypes, which can be rescued in a double knockout. However, the mechanisms by which Tsc2 and Fmr1 result in opposite synaptic phenotypes is not yet clear. In post-hoc analysis, we found that *Tsc2* gene expression was downregulated in *Fmr1*-KO blood (Welch’s *t*-test *p*-value 0.0045). Thus, in the *Tsc2+/− Fmr1*-KO mice, the Tsc2 expression level may be closer to WT and may contribute to the rescue of the synaptic physiology. This finding points to another level of interaction between the FMRP and Tsc2 functions in the cell. Future studies are required to understand whether *Fmr1* loss leads to changes in Tsc2 mRNA via transcriptional or post-transcriptional regulation.

Similarities between the proposed roles of TSC1/2 and FMRP proteins in regulation of protein synthesis have led to the popular hypothesis that hyperactive mTOR signaling is pathogenic in both FXS and TSC. Our results indicate the gene expression dysregulation differs markedly in the two conditions. This is consistent with the previously reported changes in neuronal morphology in each of these models. Neurons deficient in *Tsc1* or *Tsc2* display lower dendritic spine density in contrast to *Fmr1*-KO neurons [50], which have increased spine density [51]. At the biochemical level there are also some important differences. In *TSC1/2*-null cells, mTORC1-dependent negative feedback mechanisms exist to dampen the activation of upstream components of the network such that Akt activation is decreased [52]. However, in *Fmr1*-KO neurons, Akt activity is enhanced [27]. While mTORC1 may be activated because of loss of either Tsc1/2 or FMRP, the neuronal phenotype and gene expression profiles may be altered by changes in the activation of other signaling pathways. Such differences have implications for targeted treatment options of the two distinct genetic conditions.

The mouse models we have explored have been developed as models of ASD with known divergent genetic etiologies. The overall divergence in gene expression dysregulation in these two models does not rule out shared downstream effects, and indeed we observed an overlapping dysregulation of the cytokine signaling pathway. Nonetheless, it does suggest that a multiplicity of therapeutics will have to be developed for the varied mechanisms contributing to the increasingly fine-grained distinctions between the etiologies of ASD.

We could identify similar sets of biological pathways enriched in both tissues. In the *Fmr1-*KO mice, the PI3K signaling pathway was dysregulated in both blood and brain, while the ribosome pathway was dysregulated in both tissues of *Tsc2 +/−.* With the two mouse models, we also could identify biological pathways that were positively correlated with genetic background in both tissues. Previous studies demonstrated that peripheral blood expression signatures could be used to classify the clinical conditions of brain disorders [35,53]. Likewise, our results suggest that peripheral blood signatures could be used to identify genotypes as well as some transcriptional changes present in brain.

The current study is limited by the different background strains of *Fmr1*-KO and *Tsc2+/−* mice and by the small sample size. The differentially expressed genes between two WT background strains overlapped with the significant genes in each murine model – 5 for *Fmr1*-KO and 9 for *Tsc2+/−*. Although 1,486 probe sets were significantly differentially expressed between WT mice of two different backgrounds, none of these genes was a member of the significant pathways that characterized *Fmr1*-KO and *Tsc2+/−* mice in blood and brain. Due to the small sample size, we did not have enough statistical power for microarray experiments to detect and compare relatively low-expressed genes. Further study using the same background strain and increasing the sample size will be essential to confirm our findings, and using a more sensitive quantification method such as RNA-seq will improve sensitivity for low abundance transcripts. For the *Fmr1*-KO model, we did not include female mice heterozygous for *Fmr1* in this experiment. Heterozygous *Fmr1* female mice should exhibit genetic mosaicism due to random X-inactivation of one X chromosome during development. For this reason, most previous studies characterized male *Fmr1* KO models. Qin and colleagues performed an interesting comparison of male *Fmr1*-KO and homozygous and heterozygous KO in female mice [54]. They reported that only the homozygous mice had a deficit on the passive avoidance test, whereas both homozygous and heterozygous female mice exhibited hyperactivity and increased susceptibility to seizures. A follow-up experiment with both sexes and different dosages of *Fmr1* in female mice with a larger sample size would be ideal since a gender effect on global gene expression profiles should be considered when both sexes are included in the experiment.

## Conclusions

Contrary to our initial hypothesis that *Fmr1*-KO and *Tsc2+/−* mice would share a transcriptional signature, we found that the two mouse models presented distinct sets of differentially expressed genes. In retrospect, this is not surprising as multiple lines of evidence suggest that FXS and TSC are actually driven by opposite molecular phenotypes [49]. Despite these gene-level differences, however, we observed that cytokine signaling, cell communication, and hematopoietic cell lineage genes were differentially expressed in both mouse strains. Second, our results show that blood expression signatures mirror many aspects of the brain transcriptome. Specifically, several pathways were dysregulated in both the brain and blood of the two mouse models studied here. This confirmation is important for the future use of blood tissue to study neurodevelopmental disorders.

## Availability of supporting data

The data set supporting the results of this article is available in the Gene Expression Omnibus repository with the accession identifier GSE40630 (http://www.ncbi.nlm.nih.gov/geo/query/acc.cgi?acc=GSE40630).

## Abbreviations

Akt: v-Akt murine thymoma viral oncogene; ASD: Autism spectrum disorder; CA1: Cornu ammonis 1; Chd7: Chromodomain-helicase-DNA-binding protein 7; Dgkb: Diacylglycerol kinase beta; Dgkg: Diacylglycerol kinase gamma; Dgkh: Diacylglycerol kinase eta; EGF: Epidermal growth factor; EIF4E: Eukaryotic translation initiation factor 4E; EPS8: Epidermal growth factor receptor pathway substrate 8; EPS8L1: Epidermal growth factor receptor pathway substrate 8 like 1; FDR: False discovery rate; Fmr1: Fragile X mental retardation 1; FMRP: Fragile X mental retardation protein; FXS: Fragile X syndrome; Grin3a: N-methyl-D-aspartate receptor subtype 3a; GSEA: Gene set enrichment analysis; Inpp4b: Inositol polyphosphate-4-phosphatase, type II; Inpp5a: Type I inositol-1,4,5-trisphosphate 5-phosphatase; Itpr3: Inositol 1,4,5-trisphosphate receptor, type 3; KEGG: Kyoto encyclopedia of genes and genomes; KO: Knockout; mGluR: Metabotropic glutamate receptor; mTOR: Mammalian target of rapamycin; mTORC1: Mammalian target of rapamycin complex 1; Oxtr: Oxytocin receptor; PI3K: Phosphatidylinositide 3-kinases; Plcb4: 1-Phosphatidylinositol-4,5-bisphosphate phosphodiesterase beta-4; PLIER: Probe logarithmic Intensity error model; Prkca: Protein kinase C, alpha; PTEN: Phosphatase and tensin homolog; RNA-seq: mRNA sequencing; Taf1c: TATA box-binding protein-associated factor RNA polymerase I subunit C; Tmlhe: Trimethyllysine dioxygenase; TSC: Tuberous sclerosis complex; Tsc1: Tuberous sclerosis protein 1; Tsc2: Tuberous sclerosis protein 2; WT: Wild type.

## Competing interests

MFB declares a financial interest in Seaside Therapeutics. SWK, MS, CDC, MGC, JL, DK, LMK, and ISK report no biomedical financial interests. The authors declare no competing financial interests.

## Authors’ contributions

SWK and CDC collected and analyzed the data, and JDL and MHW performed qRT-PCR validation. SWK and MGC performed statistical analyses. MS provided *Tsc2* heterozygous mouse, and DK and MFB provided *Fmr1* knockout mouse. ISK and LMK conceived the study, and SWK, MS, and ISK wrote the manuscript. All authors read and approved the final manuscript.

## Supplementary Material

Additional file 1: Table S1 The 107 transcripts were differentially expressed in *Fmr1*-KO mice compared to wildtype littermates. These probesets were used for the hierarchical clustering analysis as showed in Figure 1A. The transcript that was assigned for uncharacterized gene was designated as ‘NA’ with the Affymetrix probeset identifiers (AffyID). The p-value was estimated from a linear model using genotype and tissue as predicting variables for each probeset. The q-value denotes the false discovery rate that were calculated from the distribution of p-values using Storey and Tibshirani’s (see Methods). Negative values in the fold changes represent down-regulated transcripts in transgenic mice. **Table S2.** The 115 transcripts were differentially expressed in *Tsc2* +/- mice compared to wildtype littermates. These probesets were used for the hierarchical clustering analysis as showed in Figure 1B. The transcript that was assigned for uncharacterized gene was designated as ‘NA’ with the Affymetrix probeset identifiers (AffyID). The p-value was estimated from a linear model using genotype and tissue as predicting variables for each probeset. The q-value denotes the false discovery rate that were calculated from the distribution of p-values using Storey and Tibshirani’s (see Methods). Negative values in the fold changes represent down-regulated transcripts in transgenic mice. Click here for file

## References

[B1] Scherer SW Dawson G Risk factors for autism: translating genomic discoveries into diagnostics Hum Genet 2011 130 123 148 10.1007/s00439-011-1037-2 21701786

[B2] Schaaf CP Zoghbi HY Solving the autism puzzle a few pieces at a time Neuron 2011 70 806 808 10.1016/j.neuron.2011.05.025 21658575

[B3] Abrahams BS Geschwind DH Advances in autism genetics: on the threshold of a new neurobiology Nat Rev Genet 2008 9 341 355 10.1038/nrg2346 18414403 PMC2756414

[B4] Patterson PH Maternal infection and immune involvement in autism Trends Mol Med 2011 17 389 394 10.1016/j.molmed.2011.03.001 21482187 PMC3135697

[B5] Grandjean P Landrigan PJ Developmental neurotoxicity of industrial chemicals Lancet 2006 368 2167 2178 10.1016/S0140-6736(06)69665-7 17174709

[B6] Kohane IS McMurry A Weber G MacFadden D Rappaport L Kunkel L Bickel J Wattanasin N Spence S Murphy S Churchill S The co-morbidity burden of children and young adults with autism spectrum disorders PLoS One 2012 7 e33224 10.1371/journal.pone.0033224 22511918 PMC3325235

[B7] Hagerman R Hoem G Hagerman P Fragile X and autism: intertwined at the molecular level leading to targeted treatments Mol Autism 2010 1 12 10.1186/2040-2392-1-12 20858229 PMC2954865

[B8] Harrison JE Bolton PF Annotation: tuberous sclerosis J Child Psychol Psychiatry 1997 38 603 614 10.1111/j.1469-7610.1997.tb01687.x 9315970

[B9] Silverman JL Yang M Lord C Crawley JN Behavioural phenotyping assays for mouse models of autism Nat Rev Neurosci 2010 11 490 502 10.1038/nrn2851 20559336 PMC3087436

[B10] Ey E Leblond CS Bourgeron T Behavioral profiles of mouse models for autism spectrum disorders Autism Research 2011 4 5 16 10.1002/aur.175 21328568

[B11] Nie D Di Nardo A Han JM Baharanyi H Kramvis I Huynh T Dabora S Codeluppi S Pandolfi PP Pasquale EB Sahin M Tsc2-Rheb signaling regulates EphA-mediated axon guidance Nat Neurosci 2010 13 163 172 10.1038/nn.2477 20062052 PMC2812631

[B12] Ehninger D Han S Shilyansky C Zhou Y Li W Kwiatkowski DJ Ramesh V Silva AJ Reversal of learning deficits in a Tsc2+/− mouse model of tuberous sclerosis Nat Med 2008 14 843 848 10.1038/nm1788 18568033 PMC2664098

[B13] Bear MF Therapeutic implications of the mGluR theory of fragile X mental retardation Genes Brain Behav 2005 4 393 398 10.1111/j.1601-183X.2005.00135.x 16098137

[B14] Fatemi SH Aldinger KA Ashwood P Bauman ML Blaha CD Blatt GJ Chauhan A Chauhan V Dager SR Dickson PE Estes AM Goldowitz D Heck DH Kemper TL King BH Martin LA Millen KJ Mittleman G Mosconi MW Persico AM Sweeney JA Webb SJ Welsh JP Consensus paper: pathological role of the cerebellum in autism Cerebellum 2012 11 777 807 10.1007/s12311-012-0355-9 22370873 PMC3677555

[B15] Jeste SS Sahin M Bolton P Ploubidis GB Humphrey A Characterization of autism in young children with tuberous sclerosis complex J Child Neurol 2008 23 520 525 10.1177/0883073807309788 18160549

[B16] Tsai P Sahin M Mechanisms of neurocognitive dysfunction and therapeutic considerations in tuberous sclerosis complex Curr Opin Neurol 2011 24 106 113 10.1097/WCO.0b013e32834451c4 21301339 PMC3059306

[B17] Butler MG Dasouki MJ Zhou XP Talebizadeh Z Brown M Takahashi TN Miles JH Wang CH Stratton R Pilarski R Eng C Subset of individuals with autism spectrum disorders and extreme macrocephaly associated with germline PTEN tumour suppressor gene mutations J Med Genet 2005 42 318 321 10.1136/jmg.2004.024646 15805158 PMC1736032

[B18] Redfern RE Daou MC Li L Munson M Gericke A Ross AH A mutant form of PTEN linked to autism Protein Sci 2010 19 1948 1956 10.1002/pro.483 20718038 PMC2998728

[B19] McBride KL Varga EA Pastore MT Prior TW Manickam K Atkin JF Herman GE Confirmation study of PTEN mutations among individuals with autism or developmental delays/mental retardation and macrocephaly Autism Res 2010 3 137 141 10.1002/aur.132 20533527

[B20] Cusco I Medrano A Gener B Vilardell M Gallastegui F Villa O Gonzalez E Rodriguez-Santiago B Vilella E Del Campo M Perez-Jurado LA Autism-specific copy number variants further implicate the phosphatidylinositol signaling pathway and the glutamatergic synapse in the etiology of the disorder Hum Mol Genet 2009 18 1795 1804 10.1093/hmg/ddp092 19246517 PMC2671988

[B21] Kelleher RJ Bear MF The autistic neuron: troubled translation? Cell 2008 135 401 406 10.1016/j.cell.2008.10.017 18984149

[B22] Neves-Pereira M Muller B Massie D Williams JH O'Brien PC Hughes A Shen SB Clair DS Miedzybrodzka Z Deregulation of EIF4E: a novel mechanism for autism J Med Genet 2009 46 759 765 10.1136/jmg.2009.066852 19556253

[B23] Bromley RL Mawer G Clayton-Smith J Baker GA Autism spectrum disorders following in utero exposure to antiepileptic drugs Neurology 2008 71 1923 1924 10.1212/01.wnl.0000339399.64213.1a 19047565

[B24] Gurpur PB Liu J Burkin DJ Kaufman SJ Valproic acid activates the PI3K/Akt/mTOR pathway in muscle and ameliorates pathology in a mouse model of Duchenne muscular dystrophy Am J Pathol 2009 174 999 1008 10.2353/ajpath.2009.080537 19179609 PMC2665759

[B25] Dufour-Rainfray D Vourc'h P Le Guisquet AM Garreau L Ternant D Bodard S Jaumain E Gulhan Z Belzung C Andres CR Chalon S Guilloteau D Behavior and serotonergic disorders in rats exposed prenatally to valproate: a model for autism Neurosci Lett 2010 470 55 59 10.1016/j.neulet.2009.12.054 20036713

[B26] Sharma A Hoeffer CA Takayasu Y Miyawaki T McBride SM Klann E Zukin RS Dysregulation of mTOR signaling in fragile X syndrome J Neurosci 2010 30 694 702 10.1523/JNEUROSCI.3696-09.2010 20071534 PMC3665010

[B27] Gross C Nakamoto M Yao X Chan CB Yim SY Ye K Warren ST Bassell GJ Excess phosphoinositide 3-kinase subunit synthesis and activity as a novel therapeutic target in fragile X syndrome J Neurosci 2010 30 10624 10638 10.1523/JNEUROSCI.0402-10.2010 20702695 PMC2924772

[B28] Vanderklish PW Edelman GM Differential translation and fragile X syndrome Genes Brain Behav 2005 4 360 384 10.1111/j.1601-183X.2005.00134.x 16098135

[B29] Amaral DG Schumann CM Nordahl CW Neuroanatomy of autism Trends Neurosci 2008 31 137 145 10.1016/j.tins.2007.12.005 18258309

[B30] Bauman ML Kemper TL Neuroanatomic observations of the brain in autism: a review and future directions Int J Dev Neurosci 2005 23 183 187 10.1016/j.ijdevneu.2004.09.006 15749244

[B31] Insel TR Fernald RD How the brain processes social information: searching for the social brain Annu Rev Neurosci 2004 27 697 722 10.1146/annurev.neuro.27.070203.144148 15217348

[B32] Tsai PT Hull C Chu Y Greene-Colozzi E Sadowski AR Leech JM Steinberg J Crawley JN Regehr WG Sahin M Autistic-like behaviour and cerebellar dysfunction in Purkinje cell Tsc1 mutant mice Nature 2012 488 647 651 10.1038/nature11310 22763451 PMC3615424

[B33] Huber KM Gallagher SM Warren ST Bear MF Altered synaptic plasticity in a mouse model of fragile X mental retardation Proc Natl Acad Sci USA 2002 99 7746 7750 10.1073/pnas.122205699 12032354 PMC124340

[B34] Onda H Lueck A Marks PW Warren HB Kwiatkowski DJ Tsc2(+/−) mice develop tumors in multiple sites that express gelsolin and are influenced by genetic background J Clin Invest 1999 104 687 695 10.1172/JCI7319 10491404 PMC408440

[B35] Kong SW Collins CD Shimizu-Motohashi Y Holm IA Campbell MG Lee IH Brewster SJ Hanson E Harris HK Lowe KR Saada A Mora A Madison K Hundley R Egan J McCarthy J Eran A Galdzicki M Rappaport L Kunkel LM Kohane IS Characteristics and predictive value of blood transcriptome signature in males with autism spectrum disorders PLoS One 2012 7 e49475 10.1371/journal.pone.0049475 23227143 PMC3515554

[B36] Storey JD Tibshirani R Statistical significance for genomewide studies Proc Natl Acad Sci USA 2003 100 9440 9445 10.1073/pnas.1530509100 12883005 PMC170937

[B37] Subramanian A Tamayo P Mootha VK Mukherjee S Ebert BL Gillette MA Paulovich A Pomeroy SL Golub TR Lander ES Mesirov JP Gene set enrichment analysis: a knowledge-based approach for interpreting genome-wide expression profiles Proc Natl Acad Sci USA 2005 102 15545 15550 10.1073/pnas.0506580102 16199517 PMC1239896

[B38] Basu SN Kollu R Banerjee-Basu S AutDB: a gene reference resource for autism research Nucleic Acids Res 2009 37 D832 D836 10.1093/nar/gkn835 19015121 PMC2686502

[B39] Tocchetti A Confalonieri S Scita G Di Fiore PP Betsholtz C In silico analysis of the EPS8 gene family: genomic organization, expression profile, and protein structure Genomics 2003 81 234 244 10.1016/S0888-7543(03)00002-8 12620401

[B40] Ascano M Jr Mukherjee N Bandaru P Miller JB Nusbaum JD Corcoran DL Langlois C Munschauer M Dewell S Hafner M Williams Z Ohler U Tuschl T FMRP targets distinct mRNA sequence elements to regulate protein expression Nature 2012 492 382 386 10.1038/nature11737 23235829 PMC3528815

[B41] Stamatakou E Marzo A Gibb A Salinas PC Activity-dependent spine morphogenesis: a role for the actin-capping protein Eps8 J Neurosci 2013 33 2661 2670 10.1523/JNEUROSCI.0998-12.2013 23392693 PMC3590009

[B42] Menna E Zambetti S Morini R Donzelli A Disanza A Calvigioni D Braida D Nicolini C Orlando M Fossati G Cristina Regondi M Pattini L Frassoni C Francolini M Scita G Sala M Fahnestock M Matteoli M Eps8 controls dendritic spine density and synaptic plasticity through its actin-capping activity EMBO J 2013 10.1038/emboj.2013.107 PMC3680733 23685357

[B43] Weichhart T Saemann MD The multiple facets of mTOR in immunity Trends Immunol 2009 30 218 226 10.1016/j.it.2009.02.002 19362054

[B44] Lee PS Tsang SW Moses MA Trayes-Gibson Z Hsiao LL Jensen R Squillace R Kwiatkowski DJ Rapamycin-insensitive up-regulation of MMP2 and other genes in tuberous sclerosis complex 2-deficient lymphangioleiomyomatosis-like cells Am J Respir Cell Mol Biol 2010 42 227 234 10.1165/rcmb.2009-0050OC 19395678 PMC2822984

[B45] Ehninger D Sano Y de Vries PJ Dies K Franz D Geschwind DH Kaur M Lee YS Li W Lowe JK Nakagawa JA Sahin M Smith K Whittemore V Silva AJ Gestational immune activation and Tsc2 haploinsufficiency cooperate to disrupt fetal survival and may perturb social behavior in adult mice Mol Psychiatry 2012 17 62 70 10.1038/mp.2010.115 21079609 PMC3118259

[B46] Ashwood P Nguyen DV Hessl D Hagerman RJ Tassone F Plasma cytokine profiles in Fragile X subjects: is there a role for cytokines in the pathogenesis? Brain Behav Immun 2010 24 898 902 10.1016/j.bbi.2010.01.008 20102735 PMC3626458

[B47] Yuskaitis CJ Beurel E Jope RS Evidence of reactive astrocytes but not peripheral immune system activation in a mouse model of fragile X syndrome Biochim Biophys Acta 2010 1802 1006 1012 10.1016/j.bbadis.2010.06.015 20600866 PMC2942952

[B48] Narayanan U Nalavadi V Nakamoto M Thomas G Ceman S Bassell GJ Warren ST S6K1 phosphorylates and regulates fragile X mental retardation protein (FMRP) with the neuronal protein synthesis-dependent mammalian target of rapamycin (mTOR) signaling cascade J Biol Chem 2008 283 18478 18482 10.1074/jbc.C800055200 18474609 PMC2441545

[B49] Auerbach BD Osterweil EK Bear MF Mutations causing syndromic autism define an axis of synaptic pathophysiology Nature 2011 480 63 68 10.1038/nature10658 22113615 PMC3228874

[B50] Tavazoie SF Alvarez VA Ridenour DA Kwiatkowski DJ Sabatini BL Regulation of neuronal morphology and function by the tumor suppressors Tsc1 and Tsc2 Nat Neurosci 2005 8 1727 1734 10.1038/nn1566 16286931

[B51] Comery TA Harris JB Willems PJ Oostra BA Irwin SA Weiler IJ Greenough WT Abnormal dendritic spines in fragile X knockout mice: maturation and pruning deficits Proc Natl Acad Sci U S A 1997 94 5401 5404 10.1073/pnas.94.10.5401 9144249 PMC24690

[B52] Choo AY Kim SG Vander Heiden MG Mahoney SJ Vu H Yoon SO Cantley LC Blenis J Glucose addiction of TSC null cells is caused by failed mTORC1-dependent balancing of metabolic demand with supply Mol Cell 2010 38 487 499 10.1016/j.molcel.2010.05.007 20513425 PMC2896794

[B53] Lunnon K Sattlecker M Furney SJ Coppola G Simmons A Proitsi P Lupton MK Lourdusamy A Johnston C Soininen H Kłoszewska I Mecocci P Tsolaki M Vellas B Geschwind D Lovestone S Dobson R Hodges A dNeuroMed Consortium A blood gene expression marker of early Alzheimer's disease J Alzheimers Dis 2013 33 737 753 23042217 10.3233/JAD-2012-121363

[B54] Qin M Kang J Smith CB A null mutation for Fmr1 in female mice: effects on regional cerebral metabolic rate for glucose and relationship to behavior Neuroscience 2005 135 999 1009 10.1016/j.neuroscience.2005.06.081 16154294

