# Supplementary material for: Divergent dysregulation of gene expression in murine models of fragile X syndrome and tuberous sclerosis
Source: Mol Autism. 2014 Feb 24;5:16. doi: 10.1186/2040-2392-5-16 (PMC3940253; doi:10.1186/2040-2392-5-16)
Supplement: Additional file 1: Table S1 — The 107 transcripts were differentially expressed in Fmr1-KO mice compared to wildtype littermates. These probesets were used for the hierarchical clustering analysis as showed in Figure 1A. The transcript that was assigned for uncharacterized gene was designated as ‘NA’ with the Affymetrix probeset identifiers (AffyID). The p-value was estimated from a linear model using genotype and tissue as predicting variables for each probeset. The q-value denotes the false discovery rate that were calculated from the distribution of p-values using Storey and Tibshirani’s (see Methods). Negative values in the fold changes represent down-regulated transcripts in transgenic mice. Table S2. The 115 transcripts were differentially expressed in Tsc2 +/- mice compared to wildtype littermates. These probesets were used for the hierarchical clustering analysis as showed in Figure 1B. The transcript that was assigned for uncharacterized gene was designated as ‘NA’ with the Affymetrix probeset identifiers (AffyID). The p-value was estimated from a linear model using genotype and tissue as predicting variables for each probeset. The q-value denotes the false discovery rate that were calculated from the distribution of p-values using Storey and Tibshirani’s (see Methods). Negative values in the fold changes represent down-regulated transcripts in transgenic mice. [file 2040-2392-5-16-S1.docx]

**Additional file 1: Table S1.** The 107 transcripts were differentially expressed in *Fmr1*-KO mice compared to wildtype littermates. These probesets were used for the hierarchical clustering analysis as showed in Figure 1A. The transcript that was assigned for uncharacterized gene was designated as ‘NA’ with the Affymetrix probeset identifiers (AffyID). The p-value was estimated from a linear model using genotype and tissue as predicting variables for each probeset. The q-value denotes the false discovery rate that were calculated from the distribution of p-values using Storey and Tibshirani’s (see Methods). Negative values in the fold changes represent down-regulated transcripts in transgenic mice.

| **AffyID** | **Gene Symbol** | **Fold change (blood)** | **Fold change (brain)** | **p-value (genotype)** | **q-value (genotype)** | **p-value (tissue)** | **q-value (tissue)** |
| --- | --- | --- | --- | --- | --- | --- | --- |
| 10599893 | *Fmr1* | -2.5 | -3.3 | 8.85E-06 | 2.91E-01 | 8.24E-03 | 2.15E-03 |
| 10338199 | *NA* | -2.7 | -1.5 | 1.09E-04 | 8.94E-01 | 2.87E-05 | 1.31E-05 |
| 10339152 | *NA* | -36.3 | -1.5 | 2.65E-04 | 9.00E-01 | 7.13E-05 | 2.95E-05 |
| 10341178 | *NA* | -2.5 | -1.2 | 6.04E-04 | 9.00E-01 | 1.36E-05 | 6.86E-06 |
| 10489961 | *Nfatc2* | -1.1 | -1.2 | 7.61E-04 | 9.00E-01 | 2.97E-08 | 4.74E-08 |
| 10366407 | *NA* | 1.5 | 1.2 | 1.08E-03 | 9.00E-01 | 4.66E-07 | 3.98E-07 |
| 10608712 | *Tmlhe* | -1.6 | -1.2 | 1.08E-03 | 9.00E-01 | 1.39E-08 | 2.80E-08 |
| 10362751 | *NA* | 1.2 | 1.3 | 1.20E-03 | 9.00E-01 | 1.22E-05 | 6.24E-06 |
| 10383479 | *Hmga1* | -1.2 | -1.1 | 1.99E-03 | 9.00E-01 | 1.72E-07 | 1.79E-07 |
| 10562439 | *Gpatch1* | -1.3 | -1.2 | 2.04E-03 | 9.00E-01 | 3.70E-07 | 3.30E-07 |
| 10344301 | *NA* | 1.5 | 3.6 | 2.13E-03 | 9.00E-01 | 2.34E-04 | 8.46E-05 |
| 10535247 | *Iqce* | -1.2 | -1.2 | 2.17E-03 | 9.00E-01 | 6.14E-07 | 4.98E-07 |
| 10339340 | *NA* | -2.8 | -1.5 | 2.64E-03 | 9.00E-01 | 1.41E-05 | 7.05E-06 |
| 10458164 | *Cdc23* | -1.3 | -1.1 | 2.67E-03 | 9.00E-01 | 1.77E-07 | 1.83E-07 |
| 10341324 | *NA* | -2.1 | -1.6 | 2.77E-03 | 9.00E-01 | 3.55E-01 | 6.42E-02 |
| 10338183 | *NA* | -2.9 | -1.7 | 2.81E-03 | 9.00E-01 | 1.00E-06 | 7.43E-07 |
| 10368083 | *Ccdc28a* | -1.4 | -1.2 | 2.95E-03 | 9.00E-01 | 3.10E-05 | 1.40E-05 |
| 10590529 | *Tmem42* | -1.3 | -1.2 | 2.97E-03 | 9.00E-01 | 4.29E-01 | 7.58E-02 |
| 10598573 | *Gm5753* | 1.3 | 1.2 | 2.98E-03 | 9.00E-01 | 8.54E-04 | 2.73E-04 |
| 10458804 | *Gm4840* | -1.2 | -1.1 | 3.08E-03 | 9.00E-01 | 1.42E-09 | 6.42E-09 |
| 10534021 | *Rimbp2* | -2.9 | -1.2 | 3.23E-03 | 9.00E-01 | 3.27E-09 | 1.05E-08 |
| 10432972 | *Rarg* | -1.1 | -1.1 | 3.36E-03 | 9.00E-01 | 1.40E-09 | 6.35E-09 |
| 10344084 | *NA* | -1.8 | -2.2 | 3.36E-03 | 9.00E-01 | 9.53E-03 | 2.46E-03 |
| 10338851 | *NA* | -1.8 | -1.2 | 3.38E-03 | 9.00E-01 | 3.47E-06 | 2.11E-06 |
| 10338636 | *NA* | -3.5 | -1.5 | 3.46E-03 | 9.00E-01 | 3.19E-03 | 9.06E-04 |
| 10402325 | *Asb2* | 1.1 | 1.1 | 3.48E-03 | 9.00E-01 | 1.47E-08 | 2.90E-08 |
| 10547100 | *Plxnd1* | -1.2 | -1.2 | 3.55E-03 | 9.00E-01 | 5.06E-04 | 1.69E-04 |
| 10581036 | *Tk2* | -1.3 | -1.1 | 3.61E-03 | 9.00E-01 | 2.99E-01 | 5.54E-02 |
| 10370610 | *Polrmt* | -1.2 | -1.0 | 3.66E-03 | 9.00E-01 | 4.63E-10 | 3.38E-09 |
| 10484658 | *Olfr1109* | 1.4 | 1.3 | 3.84E-03 | 9.00E-01 | 1.37E-03 | 4.18E-04 |
| 10410211 | *Zfp640* | 4.1 | 2.0 | 3.86E-03 | 9.00E-01 | 5.77E-04 | 1.91E-04 |
| 10382022 | *Taco1* | -1.3 | -1.2 | 3.95E-03 | 9.00E-01 | 2.06E-06 | 1.36E-06 |
| 10549655 | *Eps8r1* | -1.4 | -1.1 | 4.00E-03 | 9.00E-01 | 6.79E-01 | 1.14E-01 |
| 10359504 | *Dnm3os* | -2.3 | -1.1 | 4.13E-03 | 9.00E-01 | 3.25E-09 | 1.05E-08 |
| 10439960 | *Cep97* | -1.4 | -1.1 | 4.24E-03 | 9.00E-01 | 2.03E-07 | 2.04E-07 |
| 10341127 | *NA* | -1.5 | -1.2 | 4.25E-03 | 9.00E-01 | 9.84E-07 | 7.30E-07 |
| 10584580 | *NA* | 1.2 | 1.3 | 4.27E-03 | 9.00E-01 | 1.78E-07 | 1.83E-07 |
| 10565532 | *Ankrd42* | -1.7 | -1.1 | 4.29E-03 | 9.00E-01 | 8.52E-10 | 4.66E-09 |
| 10342494 | *NA* | -2.0 | -1.8 | 4.31E-03 | 9.00E-01 | 3.14E-01 | 5.78E-02 |
| 10340778 | *NA* | -29.2 | -2.1 | 4.36E-03 | 9.00E-01 | 5.14E-05 | 2.20E-05 |
| 10607838 | *NA* | 1.4 | 1.3 | 4.45E-03 | 9.00E-01 | 5.12E-02 | 1.13E-02 |
| 10341092 | *NA* | -4.1 | -2.4 | 4.80E-03 | 9.00E-01 | 1.48E-04 | 5.63E-05 |
| 10444098 | *Wdr46* | -1.1 | -1.2 | 4.94E-03 | 9.00E-01 | 4.08E-07 | 3.57E-07 |
| 10341739 | *NA* | -1.8 | -1.6 | 4.99E-03 | 9.00E-01 | 3.41E-03 | 9.63E-04 |
| 10508190 | *AU040320* | -1.3 | -1.1 | 5.07E-03 | 9.00E-01 | 3.17E-07 | 2.92E-07 |
| 10342133 | *NA* | -1.5 | -1.2 | 5.11E-03 | 9.00E-01 | 2.03E-02 | 4.89E-03 |
| 10599686 | *Zfp449* | -2.6 | -1.2 | 5.19E-03 | 9.00E-01 | 1.84E-08 | 3.38E-08 |
| 10503196 | *Chd7* | -1.3 | -1.1 | 5.24E-03 | 9.00E-01 | 7.74E-12 | 3.83E-10 |
| 10567608 | *Cog7* | -1.4 | -1.2 | 5.34E-03 | 9.00E-01 | 5.14E-09 | 1.42E-08 |
| 10341060 | *NA* | -1.9 | -1.3 | 5.35E-03 | 9.00E-01 | 2.71E-04 | 9.67E-05 |
| 10380560 | *Zfp652* | -1.1 | -1.1 | 5.43E-03 | 9.00E-01 | 1.02E-05 | 5.31E-06 |
| 10343007 | *NA* | -3.5 | -1.3 | 5.50E-03 | 9.00E-01 | 4.10E-04 | 1.40E-04 |
| 10441038 | *Hlcs* | -1.3 | -1.2 | 5.50E-03 | 9.00E-01 | 9.70E-07 | 7.22E-07 |
| 10473981 | *Ambra1* | -1.1 | -1.1 | 5.51E-03 | 9.00E-01 | 2.33E-08 | 3.99E-08 |
| 10341001 | *NA* | -2.1 | -1.7 | 5.61E-03 | 9.00E-01 | 1.31E-03 | 4.03E-04 |
| 10343600 | *NA* | -2.4 | -1.5 | 5.67E-03 | 9.00E-01 | 1.06E-03 | 3.31E-04 |
| 10432139 | *Zfp641* | -1.2 | -1.1 | 5.80E-03 | 9.00E-01 | 2.06E-08 | 3.65E-08 |
| 10338595 | *NA* | 1.3 | 1.3 | 5.86E-03 | 9.00E-01 | 6.91E-01 | 1.15E-01 |
| 10339846 | *NA* | -2.8 | -1.3 | 6.00E-03 | 9.00E-01 | 4.17E-06 | 2.46E-06 |
| 10392388 | *Prkca* | -1.2 | -1.2 | 6.14E-03 | 9.00E-01 | 5.38E-07 | 4.46E-07 |
| 10395606 | *NA* | -2.9 | -1.2 | 6.19E-03 | 9.00E-01 | 1.71E-06 | 1.16E-06 |
| 10449631 | *Btbd9* | -1.2 | -1.1 | 6.31E-03 | 9.00E-01 | 1.04E-09 | 5.34E-09 |
| 10504918 | *Zfp189* | -1.2 | -1.1 | 6.33E-03 | 9.00E-01 | 1.95E-10 | 2.10E-09 |
| 10371271 | *Zfp781* | -1.2 | -1.1 | 6.43E-03 | 9.00E-01 | 1.63E-11 | 5.42E-10 |
| 10488322 | *Ralgapa2* | -1.2 | -1.1 | 6.46E-03 | 9.00E-01 | 6.06E-02 | 1.32E-02 |
| 10488709 | *8430427H17Rik* | -1.2 | -1.2 | 6.52E-03 | 9.00E-01 | 1.00E-02 | 2.57E-03 |
| 10343569 | *NA* | -1.2 | -1.2 | 6.54E-03 | 9.00E-01 | 2.35E-07 | 2.29E-07 |
| 10341371 | *NA* | -1.9 | -1.5 | 6.68E-03 | 9.00E-01 | 9.28E-05 | 3.71E-05 |
| 10384229 | *NA* | 1.6 | 1.7 | 6.70E-03 | 9.00E-01 | 1.95E-02 | 4.71E-03 |
| 10531675 | *Sec31a* | -1.2 | -1.1 | 6.72E-03 | 9.00E-01 | 1.38E-07 | 1.51E-07 |
| 10551828 | *Gm5113* | -4.5 | -1.1 | 6.78E-03 | 9.00E-01 | 2.24E-09 | 8.36E-09 |
| 10381939 | *Tanc2* | -1.2 | -1.1 | 6.80E-03 | 9.00E-01 | 3.82E-10 | 3.07E-09 |
| 10340423 | *NA* | -1.2 | -1.2 | 6.83E-03 | 9.00E-01 | 2.13E-06 | 1.39E-06 |
| 10359386 | *Dars2* | -1.3 | -1.1 | 6.92E-03 | 9.00E-01 | 1.62E-08 | 3.11E-08 |
| 10462330 | *Ppapdc2* | -1.2 | -1.2 | 6.98E-03 | 9.00E-01 | 6.54E-06 | 3.62E-06 |
| 10491252 | *Samd7* | 1.2 | 1.1 | 7.19E-03 | 9.00E-01 | 9.03E-01 | 1.45E-01 |
| 10341282 | *NA* | -1.4 | -1.3 | 7.26E-03 | 9.00E-01 | 1.12E-04 | 4.39E-05 |
| 10355479 | *Ankar* | 1.8 | 1.2 | 7.27E-03 | 9.00E-01 | 2.00E-04 | 7.38E-05 |
| 10479087 | *Stx16* | -1.1 | -1.2 | 7.42E-03 | 9.00E-01 | 7.20E-07 | 5.66E-07 |
| 10528507 | *Pus7* | -1.3 | -1.2 | 7.49E-03 | 9.00E-01 | 2.53E-06 | 1.61E-06 |
| 10461782 | *Gm5512* | -1.2 | -1.2 | 7.50E-03 | 9.00E-01 | 9.97E-06 | 5.22E-06 |
| 10424905 | *Scx* | -2.0 | -1.1 | 7.54E-03 | 9.00E-01 | 8.02E-10 | 4.50E-09 |
| 10561527 | *Actn4* | -1.2 | -1.1 | 7.67E-03 | 9.00E-01 | 3.84E-08 | 5.70E-08 |
| 10442139 | *Vmn1r224* | 1.3 | 1.2 | 7.69E-03 | 9.00E-01 | 6.84E-03 | 1.82E-03 |
| 10423731 | *NA* | -3.0 | -1.3 | 7.69E-03 | 9.00E-01 | 2.54E-07 | 2.44E-07 |
| 10497920 | *Ankrd50* | -1.2 | -1.1 | 7.90E-03 | 9.00E-01 | 8.94E-11 | 1.36E-09 |
| 10342010 | *NA* | -2.0 | -1.2 | 7.93E-03 | 9.00E-01 | 1.48E-05 | 7.36E-06 |
| 10584777 | *Ddx6* | -1.1 | -1.2 | 7.95E-03 | 9.00E-01 | 1.24E-04 | 4.80E-05 |
| 10445729 | *Tcfeb* | -1.2 | -1.1 | 7.96E-03 | 9.00E-01 | 3.07E-05 | 1.40E-05 |
| 10584200 | *Rpusd4* | -1.2 | -1.0 | 8.17E-03 | 9.00E-01 | 6.69E-01 | 1.12E-01 |
| 10591224 | *Zfp560* | -3.1 | -1.2 | 8.32E-03 | 9.00E-01 | 2.81E-06 | 1.76E-06 |
| 10339227 | *NA* | -1.6 | -1.3 | 8.37E-03 | 9.00E-01 | 7.93E-06 | 4.28E-06 |
| 10343451 | *NA* | -2.1 | -1.3 | 8.40E-03 | 9.00E-01 | 2.16E-04 | 7.89E-05 |
| 10445373 | *NA* | -1.1 | -1.1 | 8.40E-03 | 9.00E-01 | 4.73E-08 | 6.67E-08 |
| 10524284 | *Ttc28* | -1.2 | -1.2 | 8.56E-03 | 9.00E-01 | 3.99E-01 | 7.12E-02 |
| 10342410 | *NA* | -1.3 | -1.4 | 8.83E-03 | 9.00E-01 | 2.01E-04 | 7.41E-05 |
| 10484577 | *Olfr1049* | 2.4 | 1.4 | 9.09E-03 | 9.00E-01 | 6.41E-02 | 1.39E-02 |
| 10342983 | *NA* | -1.5 | -1.1 | 9.12E-03 | 9.00E-01 | 3.81E-08 | 5.65E-08 |
| 10444637 | *D17H6S56E-3* | -1.5 | -1.2 | 9.15E-03 | 9.00E-01 | 3.12E-08 | 4.89E-08 |
| 10580100 | *Cc2d1a* | -1.3 | -1.1 | 9.18E-03 | 9.00E-01 | 1.87E-08 | 3.42E-08 |
| 10535586 | *Smurf1* | -1.3 | -1.1 | 9.30E-03 | 9.00E-01 | 1.52E-05 | 7.56E-06 |
| 10498296 | *Commd2* | -1.5 | -1.1 | 9.34E-03 | 9.00E-01 | 3.67E-09 | 1.14E-08 |
| 10404030 | *Hist1h3f* | 1.5 | 1.3 | 9.55E-03 | 9.00E-01 | 1.25E-03 | 3.86E-04 |
| 10501007 | *Bclp2* | -1.9 | -1.4 | 9.74E-03 | 9.00E-01 | 8.29E-07 | 6.34E-07 |
| 10340990 | *NA* | -1.2 | -1.2 | 9.86E-03 | 9.00E-01 | 2.68E-07 | 2.55E-07 |
| 10338744 | *NA* | -4.1 | -1.8 | 9.95E-03 | 9.00E-01 | 1.39E-04 | 5.32E-05 |
| 10490972 | *Trim55* | 1.3 | 1.1 | 9.99E-03 | 9.00E-01 | 3.92E-02 | 8.90E-03 |

**Additional file 1: Table S2.** The 115 transcripts were differentially expressed in *Tsc2 +/-* mice compared to wildtype littermates. These probesets were used for the hierarchical clustering analysis as showed in Figure 1B. The transcript that was assigned for uncharacterized gene was designated as ‘NA’ with the Affymetrix probeset identifiers (AffyID). The p-value was estimated from a linear model using genotype and tissue as predicting variables for each probeset. The q-value denotes the false discovery rate that were calculated from the distribution of p-values using Storey and Tibshirani’s (see Methods). Negative values in the fold changes represent down-regulated transcripts in transgenic mice.

| **AffyID** | **Gene Symbol** | **Fold change (blood)** | **Fold change (brain)** | **p-value (genotype)** | **q-value (genotype)** | **p-value (tissue)** | **q-value (tissue)** |
| --- | --- | --- | --- | --- | --- | --- | --- |
| 10383575 | *Tbcd* | -1.1 | -1.1 | 2.83E-04 | 9.28E-01 | 3.04E-06 | 2.26E-05 |
| 10533945 | *Ubc* | 1.1 | 1.1 | 4.11E-04 | 9.28E-01 | 1.55E-05 | 5.30E-05 |
| 10551966 | *Hspb6* | -1.2 | -1.2 | 5.84E-04 | 9.28E-01 | 3.37E-02 | 1.48E-02 |
| 10346116 | *NA* | -1.3 | -1.1 | 5.91E-04 | 9.28E-01 | 4.77E-07 | 1.13E-05 |
| 10474448 | *NA* | 3.9 | 1.6 | 8.20E-04 | 9.28E-01 | 1.81E-02 | 8.91E-03 |
| 10431585 | *Chkb* | 1.2 | 1.1 | 8.35E-04 | 9.28E-01 | 1.40E-05 | 4.97E-05 |
| 10339512 | *NA* | 1.4 | 1.5 | 8.39E-04 | 9.28E-01 | 7.25E-03 | 4.21E-03 |
| 10432431 | *Fam186b* | 1.1 | 1.1 | 1.06E-03 | 9.28E-01 | 1.60E-03 | 1.27E-03 |
| 10339017 | *NA* | -1.4 | -1.8 | 1.08E-03 | 9.28E-01 | 3.29E-04 | 3.87E-04 |
| 10341456 | *NA* | -2.1 | -1.6 | 1.14E-03 | 9.28E-01 | 3.74E-03 | 2.47E-03 |
| 10409924 | *Cts8* | 1.2 | 1.5 | 1.56E-03 | 9.28E-01 | 1.51E-03 | 1.22E-03 |
| 10420114 | *Tgm1* | 1.2 | 1.2 | 1.67E-03 | 9.28E-01 | 1.56E-03 | 1.25E-03 |
| 10339220 | *NA* | 2.6 | 2.2 | 1.83E-03 | 9.28E-01 | 1.26E-02 | 6.62E-03 |
| 10342492 | *NA* | -1.5 | -1.9 | 1.84E-03 | 9.28E-01 | 5.91E-01 | 1.58E-01 |
| 10363917 | *Gm5778* | 1.3 | 1.3 | 2.03E-03 | 9.28E-01 | 1.72E-01 | 5.70E-02 |
| 10524790 | *Cit* | -1.6 | -1.1 | 2.06E-03 | 9.28E-01 | 1.23E-06 | 1.52E-05 |
| 10574511 | *NA* | 1.2 | 1.2 | 2.12E-03 | 9.28E-01 | 3.97E-02 | 1.70E-02 |
| 10340560 | *NA* | -2.8 | -2.0 | 2.23E-03 | 9.28E-01 | 3.46E-01 | 1.02E-01 |
| 10372175 | *NA* | 8.8 | 1.4 | 2.42E-03 | 9.28E-01 | 4.38E-06 | 2.66E-05 |
| 10607332 | *NA* | -2.0 | -1.6 | 2.44E-03 | 9.28E-01 | 4.29E-01 | 1.22E-01 |
| 10524878 | *Vsig10* | 1.2 | 1.2 | 2.46E-03 | 9.28E-01 | 1.42E-02 | 7.32E-03 |
| 10542395 | *NA* | -1.3 | -1.3 | 2.59E-03 | 9.28E-01 | 6.86E-01 | 1.78E-01 |
| 10338940 | *NA* | -1.8 | -2.2 | 2.82E-03 | 9.28E-01 | 2.49E-01 | 7.77E-02 |
| 10530369 | *NA* | 1.3 | 1.2 | 2.86E-03 | 9.28E-01 | 4.61E-01 | 1.29E-01 |
| 10584424 | *Olfr909* | -1.3 | -1.4 | 2.86E-03 | 9.28E-01 | 3.10E-03 | 2.14E-03 |
| 10518096 | *Fhad1* | 1.4 | 1.2 | 2.93E-03 | 9.28E-01 | 5.87E-01 | 1.57E-01 |
| 10518967 | *NA* | 1.2 | 1.4 | 3.07E-03 | 9.28E-01 | 3.74E-02 | 1.62E-02 |
| 10566613 | *NA* | 1.7 | 1.7 | 3.08E-03 | 9.28E-01 | 3.03E-03 | 2.10E-03 |
| 10519314 | *NA* | 1.8 | 1.9 | 3.09E-03 | 9.28E-01 | 2.92E-02 | 1.32E-02 |
| 10342112 | *NA* | -1.5 | -1.3 | 3.11E-03 | 9.28E-01 | 7.46E-02 | 2.86E-02 |
| 10555762 | *Olfr584* | 1.2 | 1.4 | 3.16E-03 | 9.28E-01 | 7.68E-05 | 1.43E-04 |
| 10430851 | *Cyp2d22* | 1.3 | 1.1 | 3.26E-03 | 9.28E-01 | 1.28E-07 | 7.14E-06 |
| 10597770 | *NA* | 1.2 | 1.2 | 3.53E-03 | 9.28E-01 | 3.58E-02 | 1.56E-02 |
| 10391567 | *Tmem101* | -1.2 | -1.1 | 3.62E-03 | 9.28E-01 | 4.77E-06 | 2.78E-05 |
| 10511999 | *Gm136* | 1.7 | 1.3 | 3.95E-03 | 9.28E-01 | 5.76E-01 | 1.55E-01 |
| 10579181 | *Rfxank* | -1.2 | -1.1 | 3.96E-03 | 9.28E-01 | 4.64E-05 | 1.03E-04 |
| 10549655 | *Eps8r1* | 2.4 | 1.8 | 3.97E-03 | 9.28E-01 | 7.22E-02 | 2.78E-02 |
| 10340183 | *NA* | 1.5 | 1.5 | 4.01E-03 | 9.28E-01 | 8.93E-02 | 3.31E-02 |
| 10485597 | *Depdc7* | 1.2 | 1.3 | 4.03E-03 | 9.28E-01 | 4.17E-02 | 1.77E-02 |
| 10343962 | *NA* | 1.5 | 1.4 | 4.15E-03 | 9.28E-01 | 1.48E-02 | 7.57E-03 |
| 10344302 | *NA* | -1.3 | -1.3 | 4.18E-03 | 9.28E-01 | 8.77E-04 | 8.01E-04 |
| 10424894 | *Heatr7a* | 1.3 | 1.1 | 4.33E-03 | 9.28E-01 | 9.51E-07 | 1.41E-05 |
| 10345197 | *4930521A18Rik* | 1.3 | 1.7 | 4.40E-03 | 9.28E-01 | 8.30E-01 | 2.07E-01 |
| 10471739 | *Olfr338* | -1.6 | -1.3 | 4.59E-03 | 9.28E-01 | 4.11E-02 | 1.74E-02 |
| 10429641 | *Naprt1* | 1.1 | 1.3 | 4.75E-03 | 9.28E-01 | 7.49E-01 | 1.91E-01 |
| 10344094 | *NA* | 3.8 | 2.8 | 4.84E-03 | 9.28E-01 | 3.21E-01 | 9.57E-02 |
| 10484543 | *Olfr1006* | 1.2 | 1.5 | 4.84E-03 | 9.28E-01 | 1.35E-03 | 1.12E-03 |
| 10587383 | *Cd109* | 1.4 | 1.3 | 4.85E-03 | 9.28E-01 | 1.98E-01 | 6.41E-02 |
| 10470125 | *Fcna* | 1.1 | 1.3 | 4.88E-03 | 9.28E-01 | 2.79E-03 | 1.97E-03 |
| 10338356 | *NA* | 1.4 | 1.4 | 5.00E-03 | 9.28E-01 | 1.04E-01 | 3.77E-02 |
| 10338936 | *NA* | -1.7 | -1.3 | 5.04E-03 | 9.28E-01 | 1.49E-04 | 2.23E-04 |
| 10342703 | *NA* | -2.0 | -2.1 | 5.06E-03 | 9.28E-01 | 1.66E-02 | 8.30E-03 |
| 10342605 | *NA* | 3.2 | 1.6 | 5.21E-03 | 9.28E-01 | 1.18E-03 | 1.01E-03 |
| 10489349 | *Gtsf1l* | -1.1 | -1.2 | 5.57E-03 | 9.28E-01 | 2.53E-03 | 1.82E-03 |
| 10485687 | *Mett5d1* | -1.3 | -1.2 | 5.73E-03 | 9.28E-01 | 5.81E-01 | 1.56E-01 |
| 10343178 | *NA* | -1.2 | -1.4 | 5.85E-03 | 9.28E-01 | 2.04E-05 | 6.21E-05 |
| 10388310 | *Rap1gap2* | 1.4 | 1.1 | 5.91E-03 | 9.28E-01 | 4.20E-06 | 2.63E-05 |
| 10565577 | *Fam181b* | 1.4 | 1.2 | 5.94E-03 | 9.28E-01 | 6.06E-06 | 3.16E-05 |
| 10466814 | *Foxd4* | 1.2 | 1.4 | 6.01E-03 | 9.28E-01 | 8.54E-03 | 4.81E-03 |
| 10476056 | *AU015228* | 1.4 | 1.3 | 6.03E-03 | 9.28E-01 | 6.52E-02 | 2.55E-02 |
| 10427665 | *NA* | 1.2 | 1.2 | 6.09E-03 | 9.28E-01 | 1.84E-01 | 6.04E-02 |
| 10538582 | *Vmn1r15* | 1.5 | 1.5 | 6.10E-03 | 9.28E-01 | 2.27E-01 | 7.19E-02 |
| 10495867 | *Gm9372* | 1.3 | 1.3 | 6.14E-03 | 9.28E-01 | 6.17E-04 | 6.14E-04 |
| 10402415 | *Serpina11* | 1.3 | 1.2 | 6.20E-03 | 9.28E-01 | 8.83E-01 | 2.17E-01 |
| 10419426 | *Olfr722* | 1.6 | 1.3 | 6.22E-03 | 9.28E-01 | 7.46E-01 | 1.91E-01 |
| 10339590 | *NA* | -1.1 | -1.2 | 6.37E-03 | 9.28E-01 | 8.27E-01 | 2.06E-01 |
| 10590933 | *Piwil4* | 1.2 | 1.3 | 6.70E-03 | 9.28E-01 | 3.55E-01 | 1.04E-01 |
| 10441952 | *2210404J11Rik* | 1.6 | 1.2 | 6.72E-03 | 9.28E-01 | 6.99E-03 | 4.09E-03 |
| 10532332 | *Pgam5* | -1.1 | -1.1 | 6.90E-03 | 9.28E-01 | 1.06E-05 | 4.24E-05 |
| 10460831 | *NA* | -1.5 | -1.2 | 7.03E-03 | 9.28E-01 | 3.37E-05 | 8.46E-05 |
| 10395356 | *Agr3* | -1.7 | -1.1 | 7.15E-03 | 9.28E-01 | 1.77E-03 | 1.37E-03 |
| 10449163 | *Pigq* | -1.1 | -1.1 | 7.17E-03 | 9.28E-01 | 4.69E-05 | 1.03E-04 |
| 10546829 | *Oxtr* | 1.1 | 1.2 | 7.19E-03 | 9.28E-01 | 5.26E-04 | 5.45E-04 |
| 10551614 | *Ech1* | 1.5 | 1.3 | 7.24E-03 | 9.28E-01 | 2.49E-03 | 1.80E-03 |
| 10588201 | *NA* | -1.3 | -1.5 | 7.25E-03 | 9.28E-01 | 1.08E-04 | 1.79E-04 |
| 10436978 | *Cbr3* | 1.2 | 1.2 | 7.35E-03 | 9.28E-01 | 3.34E-03 | 2.27E-03 |
| 10555135 | *Gdpd4* | 1.1 | 1.2 | 7.51E-03 | 9.28E-01 | 9.29E-01 | 2.26E-01 |
| 10344609 | *NA* | -1.6 | -2.9 | 7.58E-03 | 9.28E-01 | 2.57E-01 | 7.98E-02 |
| 10366649 | *NA* | -1.2 | -1.2 | 7.61E-03 | 9.28E-01 | 9.59E-01 | 2.31E-01 |
| 10514333 | *NA* | 1.2 | 1.3 | 7.63E-03 | 9.28E-01 | 2.87E-01 | 8.74E-02 |
| 10375880 | *Nhp2* | 1.2 | 1.1 | 7.73E-03 | 9.28E-01 | 3.23E-01 | 9.64E-02 |
| 10338305 | *NA* | -2.0 | -1.5 | 7.74E-03 | 9.28E-01 | 5.01E-01 | 1.38E-01 |
| 10542340 | *8430419L09Rik* | -1.5 | -1.3 | 7.80E-03 | 9.28E-01 | 2.36E-05 | 6.76E-05 |
| 10545409 | *Vamp8* | 1.1 | 1.2 | 8.04E-03 | 9.28E-01 | 5.25E-04 | 5.44E-04 |
| 10432133 | *Olfr286* | 1.3 | 1.2 | 8.05E-03 | 9.28E-01 | 2.52E-05 | 7.09E-05 |
| 10607585 | *NA* | 1.2 | 1.3 | 8.08E-03 | 9.28E-01 | 5.37E-03 | 3.31E-03 |
| 10497033 | *Lrriq3* | 1.3 | 1.1 | 8.19E-03 | 9.28E-01 | 5.56E-01 | 1.50E-01 |
| 10589596 | *Ccdc12* | 1.2 | 1.1 | 8.21E-03 | 9.28E-01 | 4.88E-04 | 5.16E-04 |
| 10383991 | *NA* | -1.2 | -1.1 | 8.27E-03 | 9.28E-01 | 1.50E-05 | 5.20E-05 |
| 10598827 | *NA* | 1.2 | 1.1 | 8.33E-03 | 9.28E-01 | 1.39E-02 | 7.16E-03 |
| 10582129 | *Taf1c* | -1.1 | -1.1 | 8.44E-03 | 9.28E-01 | 9.58E-05 | 1.65E-04 |
| 10341939 | *NA* | 1.6 | 1.2 | 8.47E-03 | 9.28E-01 | 1.03E-02 | 5.60E-03 |
| 10456516 | *NA* | 1.2 | 1.3 | 8.51E-03 | 9.28E-01 | 3.00E-02 | 1.35E-02 |
| 10347491 | *Wnt6* | 1.1 | 1.2 | 8.57E-03 | 9.28E-01 | 1.30E-04 | 2.04E-04 |
| 10482237 | *Nr5a1* | 1.1 | 1.3 | 8.75E-03 | 9.28E-01 | 3.48E-04 | 4.03E-04 |
| 10343058 | *NA* | -1.5 | -1.5 | 8.81E-03 | 9.28E-01 | 2.59E-01 | 8.01E-02 |
| 10398859 | *Adssl1* | 1.7 | 1.2 | 8.83E-03 | 9.28E-01 | 1.09E-02 | 5.86E-03 |
| 10388282 | *NA* | 1.2 | 1.3 | 8.88E-03 | 9.28E-01 | 3.40E-01 | 1.00E-01 |
| 10519642 | *Gm4959* | 1.2 | 1.3 | 8.90E-03 | 9.28E-01 | 6.33E-01 | 1.67E-01 |
| 10387201 | *Arhgef15* | -1.1 | -1.1 | 9.04E-03 | 9.28E-01 | 1.09E-07 | 6.81E-06 |
| 10582196 | *1190005I06Rik* | 1.2 | 1.2 | 9.13E-03 | 9.28E-01 | 4.20E-05 | 9.67E-05 |
| 10343198 | *NA* | 1.1 | 1.3 | 9.15E-03 | 9.28E-01 | 5.20E-01 | 1.43E-01 |
| 10473626 | *Olfr1258* | 1.5 | 1.2 | 9.16E-03 | 9.28E-01 | 9.50E-01 | 2.29E-01 |
| 10343275 | *NA* | -1.3 | -1.4 | 9.20E-03 | 9.28E-01 | 7.68E-04 | 7.25E-04 |
| 10342889 | *NA* | 1.6 | 1.2 | 9.36E-03 | 9.28E-01 | 5.89E-05 | 1.20E-04 |
| 10341199 | *NA* | -1.2 | -1.3 | 9.40E-03 | 9.28E-01 | 1.24E-03 | 1.04E-03 |
| 10349809 | *Rbbp5* | -1.1 | -1.1 | 9.42E-03 | 9.28E-01 | 9.58E-04 | 8.59E-04 |
| 10572083 | *NA* | -1.2 | -1.4 | 9.42E-03 | 9.28E-01 | 9.12E-02 | 3.37E-02 |
| 10433179 | *Mucl1* | 1.3 | 1.4 | 9.50E-03 | 9.28E-01 | 3.24E-02 | 1.43E-02 |
| 10409063 | *NA* | -1.1 | -1.2 | 9.51E-03 | 9.28E-01 | 5.59E-01 | 1.51E-01 |
| 10339606 | *NA* | 1.8 | 2.0 | 9.54E-03 | 9.28E-01 | 2.43E-01 | 7.60E-02 |
| 10389835 | *Kif2b* | 1.2 | 1.2 | 9.58E-03 | 9.28E-01 | 9.67E-04 | 8.66E-04 |
| 10528236 | *NA* | -1.4 | -1.3 | 9.67E-03 | 9.28E-01 | 5.62E-03 | 3.43E-03 |
| 10341216 | *NA* | -1.7 | -1.7 | 9.76E-03 | 9.28E-01 | 2.62E-03 | 1.87E-03 |
| 10491895 | *NA* | -1.3 | -1.2 | 9.82E-03 | 9.28E-01 | 2.83E-02 | 1.29E-02 |
